# Fractional CO_2_ Laser Pretreatment Facilitates Transdermal Delivery of Two Vitamin C Derivatives

**DOI:** 10.3390/molecules21111547

**Published:** 2016-11-16

**Authors:** Chien-Yu Hsiao, Hsin-Ching Sung, Sindy Hu, Yau-Li Huang, Chun-Hsun Huang

**Affiliations:** 1 Department of Nutrition and Health Sciences, Chang Gung University of Science and Technology, Taoyuan 333, Taiwan; chienyuhsiaoc@sina.com; 2 Research Center for Industry of Human Ecology and Research Center for Chinese Herbal Medicine, College of Human Ecology, Chang Gung University of Science and Technology, Taoyuan 33301, Taiwan; 3 Aesthetic Medical Center, Department of Dermatology, Chang Gung Memorial Hospital, Taoyuan 333, Taiwan; hsinchingsungs@sina.com (H.-C.S.); sindyhuh@sina.com (S.H.); yaulihuangh@sina.com, (Y.-L.H.); 4 Department of Anatomy, College of Medicine, Chang Gung University, Taoyuan 333, Taiwan; 5 College of Medicine, Chang Gung University, Taoyuan 333, Taiwan; 6 Department of Cosmetic Science, Chang Gung University of Science and Technology, Taoyuan 333, Taiwan

**Keywords:** vitamin C derivatives, fractional CO_2_ laser, transdermal delivery

## Abstract

Background: Topical vitamin C derivatives have been used to treat melasma and used as a skin whitener. The aim of this study was to compare skin histology and permeation of l-ascorbic acid 2-phosphate sesquimagnesium salt (MAP-1) and magnesium l-ascorbic acid-2-phosphate (MAP-2) after fractional CO_2_ laser pretreatment. Methods: The effect of fractional laser treatment on porcine skin was examined by scanning electron microscopy and confocal laser scanning electron microscopy. The effect of fractional CO_2_ laser treatment of different fluencies and pass numbers on transdermal flux of the two vitamin C derivatives through porcine skin was examined in vitro using a Franz diffusion chamber. Results: Fluxes of MAP-1 and MAP-2 across fractional CO_2_ laser-treated (5 W) skin were eight- to 13-fold, and 20- to 22-fold higher, respectively, than the fluxes of these compounds across intact skin. Fluxes of MAP-1 and MAP-2 across fractional CO_2_ laser-treated (9 W) skin were 14- to 19-fold, and 30- to 42-fold higher, respectively, than their fluxes across intact skin. Conclusion: Fractional CO_2_ laser treatment is an effective way of delivering vitamin C derivatives into the skin.

## 1. Introduction

Vitamin C inhibits melanogenesis, promotes collagen synthesis, and has antioxidant properties [1,2,3,4,5,6]. Due to these actions, it has been used topically to restore aged skin and as a skin whitener [4,7,8]. Vitamin C itself undergoes rapid breakdown, which makes the use of stable derivatives more practical. To reach its site of action, vitamin C and its derivatives must penetrate through the stratum corneum into the skin layers underneath. The stratum corneum is composed of a layer of cornified cells and an intracellular, lipid-rich area [9,10]. Hydrophilic compounds, like vitamin C and its derivatives, can enter the hydrophilic interior of the cells in the stratum corneum, but, unlike lipophilic compounds [11,12], cannot cross the lipid-containing region underneath and, therefore, penetrate through the outer layer of the stratum corneum very poorly after topical treatment. Laser treatment can remove the stratum corneum with little damage to the skin layers underneath [13]. Therefore, laser treatment is one way to increase permeability to hydrophilic compounds [14]. However, conventional laser treatment causes the ablation of the entire skin surface, and a period of several days is required for the skin surface to recover fully [15]. Fractional laser treatment is a non-invasive treatment that uses a device to deliver a laser beam divided into thousands of microscopic treatment zones (“micropores”) that target a fraction of the skin at a time. Fractional laser treatment has been used to increase topical absorption of a number of compounds, including vitamin C derivatives [11,14,15,16,17].

CO_2_ and Er:YAG lasers have both been used to increase skin permeability. Both lasers ablate the stratum corneum, but the CO_2_ laser also produces thermal effects below the stratum corneum that can enhance transdermal delivery [18,19,20,21,22]. Our early comparison between the effects of conventional Er:YAG and CO_2_ laser pre-treatment on transdermal delivery of two vitamin C derivatives, 2-*O*-ethyl ascorbic acid and ascorbic acid 2-glucoside, showed that the two derivatives differed in their responsiveness to laser pre-treatment, and that the CO_2_ laser was more effective than the Er:YAG laser on both compounds [16]. An optimal fluency for the CO_2_ laser was not determined in this study. A later comparison of the effects of Er:YAG and CO_2_ laser pre-treatment on transdermal permeability of three additional vitamin C derivatives, 2-phospho-l-scorbic acid trisodium salt, l-ascorbic acid 2-phosphate sesquimagnesium salt (MAP-1), and magnesium l-ascorbic acid-2-phosphate (MAP-2), showed that these also differed in their responsiveness to laser pre-treatment, that CO_2_ laser pre-treatment was more effective than Er:YAG laser pre-treatment, and that optimal fluency was the same (5 W) for all three derivatives [15].

Having demonstrated that conventional CO_2_ laser treatment was more effective than the conventional Er:YAG laser in increasing permeability for the five vitamin C derivatives examined, we then investigated whether fractional CO_2_ laser pre-treatment, which causes less skin damage, could equal conventional CO_2_ laser treatment in increasing transdermal permeability. In these studies, we found that four passes of the fractional CO_2_ laser caused similar permeability but less skin disruption than conventional CO_2_ laser treatment for two skin whiteners (the vitamin C derivative ascorbic acid 2-glucoside and a chemically-unrelated skin whitener, tranexamic acid) and that as fluency increased, the number of passes needed to produce results similar to those of conventional CO_2_ laser pretreatment decreased [13,17].

Comparisons between CO_2_ laser treatment-induced permeabilities of different vitamin C derivatives are incomplete. The normalized flux of the 2-phospho-l-ascorbic acid trisodium salt through CO_2_ laser pretreated porcine skin at fluencies of 5 W and 9 W has been reported to be higher than that for the magnesium derivatives MAP-1 and MAP-2, but no studies of the effect of fractional laser pretreatment on this vitamin C derivative have been done [13], and CO_2_ laser pretreatment of nude mouse skin has reported *O*-ethyl ascorbic acid to have higher fluency at 5 W and 7 W than ascorbic acid 2-glucoside, but no comparable studies have been performed on porcine skin [16].

There is no standard pretreatment fractional CO_2_ laser procedure for MAP-1 and MAP-2 delivery. In the present study, we examined permeation of these two vitamin C derivatives through porcine skin at different fluencies and pass numbers of fractional CO_2_ laser pretreatment in order to help the clinician determine the fluency and number of passes that will result in a combination of optimal permeation and minimal skin damage.

## 2. Results

### 2.1. Ultrastructure of Fractional Laser Treatment of Porcine Skin

**Figure 1:**
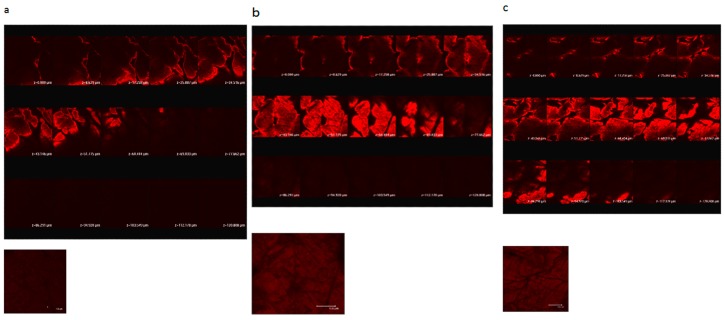
Confocal laser scanning microscopic (CLSM) micrographs of porcine skin after in vitro topical administration of rhodamine B via the skin by laser pretreatment: (**a**) Non-treatment group; (**b**) Fractional CO_2_ laser treatment at 12 mm, 5 W; (**c**) Fractional CO_2_ laser treatment at 12 mm, 9 W (original magnification, 20×). The skin specimen was viewed by CLSM at 8-μm increments through the *Z*-axis. The images below the photographs of the 15 fragments are the sum of all fragments.

Figure 1 shows confocal scanning electron microscopy of porcine skin after 5 W and 9 W fractional CO_2_ laser treatment. Untreated skin shows low permeation of the test compound used, rhodamine B, a compound with a molecular weight similar to MAP-1 and MAP-2. Fractional laser treatment causes permeation of rhodamine B, and permeation is deeper when the higher fluency is used.

**Figure 2:**
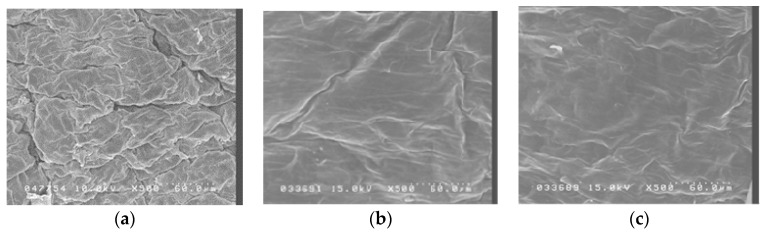
Scanning electron microscopy (SEM) observations (magnification 500×) of porcine skin. (**a**) Without any treatment; (**b**) Fractional CO_2_ laser treatment at 12 mm, 5 W; (**c**) Fractional CO_2_ laser treatment at 12 mm, 9 W.

Figure 2 shows scanning electron micrographs of porcine skin after 5 W and 9 W fractional CO_2_ laser treatment. Untreated skin (Figure 1a) shows intact, regularly arranged, overlapping corneocytes. Fractional laser treatment at 5 W and 9 W shows some irregularity, but no disruption, of the skin surface.

### 2.2. Normalized Fluxes of MAP-1 Across Porcine Skin after Fractional CO_2_ Laser Pretreatment

**Figure 3:**
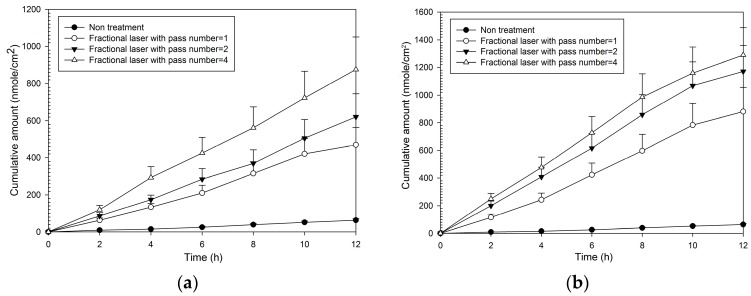
Cumulative amount of time profiles of in vitro transdermal permeation through porcine skin of MAP-1, after fractional CO_2_ laser pre-treatment with a 12 mm spot size and different pass numbers at (**a**) 5 W and (**b**) 9 W fluence.

Fluxes and enhancement ratios for MAP-1 were significantly increased compared to no treatment at each pass number at both fluencies (Table 1). Enhancement ratios for MAP-2 ranged from 8–13 at 5 W and from 14–19 at 9 W. The cumulative amount with time (Figure 3) was also increased at both fluencies compared to no laser pretreatment, and increased as fluency and pass number increased. It was only necessary to produce a small number of microscopic pores with fractional treatment to cause a large relative increase in flux, for one pass at 5 W (that is, covering only 2% of the treatment area with the microscopic pores, caused an eight-fold increase in flux compared to no treatment).

### 2.3. Normalized Fluxes of MAP-2 Across Porcine Skin after Pretreatment with a Fractional Laser

**Figure 4:**
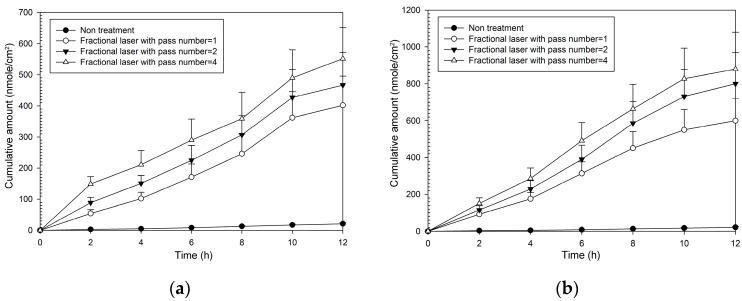
Cumulative amount of time profiles of in vitro transdermal permeation through porcine skin of MAP-2, after fractional CO_2_ laser pre-treatment with a 12 mm spot size and different pass number at (**a**) 5 W and (**b**) 9 W fluence.

As with MAP-1, all combinations of fractional laser pass number and fluency caused significantly greater flux than that seen with no fractional laser pre-treatment (Table 2). Enhancement ratios were greater than those seen with MAP-1 and ranged from 20–22 at 5 W and from 30–42 at 9 W. The pattern of the cumulative amount-time profile of MAP-2 (Figure 4) was similar with that of MAP-1. However, MAP-1 had a flux rate through untreated skin that was three times that seen with MAP-2, so although enhancement ratios were higher with MAP-2, the actual flux rate through fractional laser-treated skin was higher with MAP-1.

## 3. Discussion

Fractional CO_2_ laser treatment increased the flux through porcine skin of both MAP-1 and MAP-2, increasing as the pass number increased, and being higher at 9 W than at 5 W. The enhancement ratios seen with MAP-2 were higher than those seen with MAP-1, but the actual flux was greater with MAP-1.

This study is a continuation of our efforts to find optimal conditions to increase transdermal permeation of skin whiteners using laser pretreatment. The current results for MAP-1 and MAP-2 combined with previous results for other compounds show that fractional CO_2_ laser pretreatment at appropriate fluencies and pass numbers can produce increases in permeability equivalent to conventional pretreatment. However, they also show that the increase in permeation using fractional laser pre-treatment is very dependent of the specific compound being used, and that some puzzles remain to be investigated. Our results demonstrate such a puzzle. MAP-1 flux is slightly higher than MAP-2 flux in both fractional and conventional [13] laser-treated skin. However, when the normalized flux after conventional and fractional laser pre-treatment are compared, conventional pretreatment produces about twice the normalized flux as fractional pretreatment for MAP-1 and about four times the normalized flux as fractional pre-treatment for MAP-2 (MAP-1, approximately 185 vs. 43–70 nmol/cm^2^/h at 5 W and approximately 230 vs. 80–108 nmol/cm^2^/h at 9 W; MAP-2 approximately 210 vs. 37–42 nmol/cm^2^/h at 5 W and approximately 325 vs. 56–78 nmol/cm^2^/h at 9 W). The physical and chemical barriers in the skin that need to be crossed are the same in fractional and conventional laser pretreatment. The reasons why fractional laser treatment is less effective compared to conventional laser treatment for MAP-2 than for MAP-1 are unknown. Nor is it known why, unlike MAP-1 and MAP-2, another vitamin C derivative, ascorbic acid 2-glucoside, has a normalized flux with fractional treatment that is virtually equal (95%–100%) to that seen with conventional laser treatment [15].

A question which cannot be answered in this study because it was not performed in vivo is whether fractional CO_2_ laser treatment caused inflammation or other reactive effects. Fractional laser treatment leaves the majority of skin tissue intact and the corneocytes that remain serve as a reservoir for healing. In other studies, complete reepithelization of the SC after fractional laser treatment has been reported to be complete within one day after treatment [23,24,25,26].

The CO_2_ laser is thought to enhance the permeation of drugs in the following way. It ablates part of the SC, as can be seen in histopathological observations, an action that increases permeability by reducing the barrier function of this skin layer. It also disrupts the remnant SC structures by photomechanical waves. However coverage of the complete skin surface with conventional CO_2_ laser treatment can have undesirable thermal effects. Fractional CO_2_ laser treatment, instead of acting over the entire skin surface, uses SC ablation and photomechanical waves to introduce microscopic channels into the skin to deliver drugs into deeper skin strata in a manner similar to the microneedle technique [27].

Lasers at low fluency can promote drug delivery through precise control of SC removal. They have the advantage of not touching the skin, thus avoiding the risk of contamination, and of having a very short treatment time. Fractional laser treatment is a relatively new procedure accomplished by the placement of numerous microscopic zones of damage in the skin without injuring the surrounding skin. This laser system resurfaces only 5%–20% of the skin at one time and does not cause full epidermal wounds, so healing time is minimized. A disadvantage of laser therapy is that it takes time to determine the laser energy and drug concentration needed to deliver each specific drug through the skin.

We have previously reported on various aspects of laser-induced increases in transdermal permeability for five vitamin C derivatives, but the laser preparations, skin preparations, and objectives vary. In the current study, our goal was to find optimum fractional laser fluency and number of pass information for use in guiding clinical practice. A future useful goal might be to use the available vitamin C derivatives in identical CO_2_ laser conditions to study the influence of the specific chemical and physical characteristics of these compounds (such as molecular weight, size, polarity, pH, log partition coefficient) on permeability of porcine skin with no laser treatment, conventional laser treatment, and fractional laser treatment. The information gained would lead to a deeper understanding of the forces determining skin permeation and serve as a guide for future drug design.

## 4. Materials and Methods

### 4.1. Ascorbic Acid Derivatives

l-ascorbic acid 2-phosphate sesquimagnesium salt (MAP-1, 289.54) was obtained from Sigma-Aldrich (St. Louis, MO, USA). Magnesium l-ascorbic acid-2-phosphate (MAP-2, 759.22) was obtained from Showa Denko K.K. Company (Tokyo, Japan).

### 4.2. Laser Assembly and Experimental Protocol

The fractional CO_2_ laser used (150XJ, Sharplan Laser Inc., Yokneam, Japan) has an articulated arm and a wavelength of 10,600 nm. Its handpiece created microscopic columns of ablated skin (irradiation dots) that typically have a diameter of 150 µm (that is, an area of about 0.018 mm^2^). The treatment area of the handpiece is 12 × 12 mm and contains 160 (13 × 13) irradiation dots, covering 2% of the total treatment area when one pulse of the laser is used. The distance between the laser and the skin was 10 mm, and the duration of the laser treatment was 1~3 s. Skin temperature was not determined after laser treatment, but as can be seen in Figure 2, and experimental skin showed little damage after laser treatment.

One to four passes at fluencies of 5 W and 9 W were used in the current study. The handpiece was rotated when more than one pass was used, so that the irradiation areas did not overlap.

### 4.3. Porcine Skin Samples

Skin from eight one-week-old, pathogen-free pigs was supplied by the Animal Technology Institute Taiwan (Miaoli, Taiwan). The supplier harvested the dorsal skin after euthanizing the piglets by electrocution, cut the skin to appropriate size, and shipped it directly to the author’s laboratory. For these studies, ten 2 × 2 cm^2^ portions of dorsal skin were removed from each pig (one for each laser condition), and were further divided as needed. The skin samples were then used for scanning electron microscopy (SEM, Hitachi S-5000, Tokyo, Japan), confocal scanning electron microscopy (CLSM, Leica Microsystems, Manheim, Germany), and in vitro permeation studies of the two vitamin C derivatives. TEM, SEM, and diffusion assays were all performed on skin from the same pig. Eight pigs were used in all. Due to the many combinations and evaluations conducted in the present study, we needed at least 200 individual pieces of skin. The skin from one pig can be cut into 20–25 pieces. So eight pigs was a number to use for the study.

### 4.4. Ultrastructure Examination by Scanning Electron Microscopy

Porcine skin samples either with or without laser treatment were cut into appropriate-sized cubes, immediately fixed in 3% paraformaldehyde and 2% glutaraldehyde in 0.1 M cacodylate buffer (pH 7.4) at 4 °C overnight, then washed with 0.1 M cacodylate and 7% sucrose buffer three times for 15 min, post-fixed with 2% osmium tetroxide for 1 h, washed three times as before, and immersed for 30 min in 0.5% aqueous uranyl acetate. Specimens were then dehydrated in graded concentrations of ethanol, transferred to isoamyl acetate, and critical-point dried using liquid CO_2_. After drying, specimens were affixed with gold-palladium in an ion coater and examined with a scanning electron microscope (Hitachi S-5000, Tokyo, Japan).

### 4.5. In Vitro Permeation of Vitamin C Derivatives

To study the ability of the vitamin C derivatives to permeate skin, a section of porcine dorsal skin was mounted in a Franz side-by-side diffusion cell with the stratum corneum facing the donor compartment. The stratum corneum was then given laser treatment at specified fluencies and pass numbers, after which its surface was wiped several times with a cotton wool swab. The receptor compartment (5.5 mL) was filled with citrate-phosphate buffer (pH 7.4). The donor compartment (0.5 mL) contained 13 mM MAP-1 or MAP-2, also in a citrate-phosphate buffer (pH 7.4). The receptor compartment was maintained at 37% and its contents stirred with a magnetic bar at 600 rpm. At appropriate intervals, 300 µL aliquots were withdrawn from the receptor compartment and immediately replaced with an equal volume of fresh receptor solution. Sampling was performed for a 12 h period, and the amount of drug in the receptor medium was determined by high-performance liquid chromatography (HPLC) (Thermo Ultimate 3000LC, Thermo Fisher Scientific, Sunnyvale, CA, USA). We did not analyze the amount of MAP-1 or MAP-2 deposited on skin during these experiments. However, these vitamin C derivatives are similar in physical properties and were not expected to differ in the amount deposited.

### 4.6. HPLC Analysis of MAP-1 and MAP-2

The amount of MAP-1 or MAP-2 in the samples was analyzed using a 15-cm-long, 4.6 mm-inner diameter Inertsil ODS-4V column (GL Science, Tokyo, Japan), a UltiMate 3000 pump (Thermo Fisher Scientific), a UltiMate 3000 autosampler, and a UltiMate 3000 UV detector. The mobile phase was set at a flow rate of 1 mL/min, and the UV detector set at a wavelength of 254 nm.

### 4.7. Rhodamine B Permeation of Laser-Treated Porcine Skin

Sections of dorsal skin were exposed to fractional CO_2_ laser pretreatment. The skin samples were glued into a glass cylinder with an available area of 0.7854 cm^2^ that was filled with 0.2 mL of 0.1% rhodamine B buffer (pH 7.4) and incubated for one hour. To examine rhodamine B fluorescence, the skin thickness was optically scanned at approximately 8 µm increments through the Z-axis of a Leica TCS SP2 confocal microscope (Manheim, Germany). Optical excitation was done with a 514-nm argon laser beam and fluorescence emission was detected at 590 nm–635 nm.

### 4.8. Data

To analyze permeation, the total amount of vitamin C derivative permeating across a unit skin surface was calculated and plotted as a function of time. Flux (nmole/cm^2^/h) was calculated from the slope of the linear portion of this curve. Normalized flux was calculated by extrapolating the recorded laser-induced permeation to 100% of the area available in the no-treatment condition.

### 4.9. Statistical Analysis

Data were graphed and analyzed using SigmaPlot software (version 12, Systat Software Inc., Chicago, IL, USA). The Kolmogorov-Smirnov test was used to examine the normality of the data in each group. The results showed the data of each group to be normally distributed. Cumulative amount-time profiles of in vitro transdermal permeation were graphed as a line plot with mean and standard deviation (SD) for each condition. Results of flux across porcine skin were summarized as mean ± SD for each condition, and these results were compared using one-way analysis of variance with a post hoc Duncan test. For the sample test in a one-way ANOVA method, a sample size of eight in each group achieved 100% power to detect differences among means versus the alternative methods of testing equal means using an F test with a *p* < 0.05 significance level. Statistical assessments were considered significant at *p* < 0.05.

In conclusion, fractional CO_2_ laser pretreatment is an effective way of improving transdermal delivery of MAP-1 and MAP-2, and may have future clinical usefulness.

## Figures and Tables

**Table 1:** MAP-1 normalized fluxes across porcine skin after pretreatment with a fractional CO_2_ laser (*n* = 8 for each fluency condition).

Fluence (W)	Pass Number	Flux ‡ (nmol/cm^2^/h)	Enhancement Ratio † (ER)
0 (no treatment)	0	5.62 ± 0.89 ^a^	1
5	1	43.09 ± 5.89 ^b^	8
	2	55.65 ± 6.92 ^b^	10
	4	74.40 ± 8.16 ^c^	13
9	1	80.28 ± 11.02 ^c^	14
	2	101.15 ± 16.58 ^c^	18
	4	107.83 ± 15.22 ^c^	19

Each value is represented as mean ± SD (*n* = 8). † Enhancement ratio (ER) is the flux of the laser-pretreated group/flux of the no-treatment group, that is, fluence = 0 W and pass number = 0. NA: Not assessed. ‡ *p* < 0.05 through one-way ANOVA test with a post-hoc Duncan test a < b < c.

**Table 2:** MAP-2 normalized fluxes across porcine skin after pretreatment with a fractional CO_2_ laser (*n* = 8 for each fluency condition).

Fluence (W)	Pass Number	Flux ‡ (nmol/cm^2^/h)	Enhancement Ratio † (ER)
0 (No treatment)	0	1.86 ± 0.43 ^a^	1
5	1	37.06 ± 4.22 ^b^	20
	2	40.15 ± 5.85 ^b^	22
	4	41.72 ± 6.05 ^b^	22
9	1	56.06 ± 7.22 ^c^	30
	2	73.28 ± 8.02 ^d^	39
	4	77.71 ± 8.14 ^d^	42

Each value is represented as mean ± SD (*n* = 8). † Enhancement ratio (ER) is the flux of laser-pretreated group/flux of no-treatment group, that is, fluence = 0 W and pass number = 0. NA: Not assessed. ‡ *p* < 0.05 through one-way ANOVA test with a post-hoc Duncan test a < b < c < d.
